# Quantifying the qualities of language

**DOI:** 10.1371/journal.pone.0232198

**Published:** 2020-05-05

**Authors:** P. A. Hancock, William G. Volante

**Affiliations:** 1 Psychology Department, University of Central Florida, Orlando, Florida, United States of America; 2 Institute for Simulation and Training, Orlando, Florida, United States of America; Wright State University, UNITED STATES

## Abstract

We here quantify the qualities of language. Specifically, we derive numerical values, and associated variability scores for statements of general probability as well as for anchor terms commonly expressed on Likert-type scales. Our results show significant inter-responder variability in the understanding of such terms. We also demonstrate scale compression such that average estimates are poorly aligned to the a priori expectations of equal response spacing in commonly used verbal response instruments. Terms further show intriguing propensities such that people agree more about statements of agreement, but manifestly disagree more about statements of disagreement. The valence of these terms is thus systematically related to their degree of common understanding. The accuracy of language, especially when used in conjunction with modern advanced technologies, proves vital for effective communication. Our work demonstrates that the tool of language still remains a fairly blunt one, but one that can be sharpened by systematic quantitative evaluation, as we demonstrate here.

## Introduction

Language is one of the most powerful tools that human beings possess. In all of its various incarnations, language is the fundamental foundation for common communication between virtually all rationale agents. Language is ubiquitous across human societies. The original impetus for such a tool most probably lies in its capacity to transfer thoughts, ideas, and emotions between living organisms. As a result, language represents one of the fundamental pillars of civilization [1]. However, the tool of language is far from perfect. Often, in the course of everyday conversation, there occur problems inherent in its imprecision of expression [2,3]. Even when referring to common terms and concepts, people do not always mean exactly the same thing. Such imprecisions are further complicated by factors such as the use of different native tongues, which are themselves embedded in varying cultural contexts. Assumptions intrinsic to such different cultures further introduce the potential for linguistic confusion, as does also each speaker and listener’s own individual personality profile.

Such variability, while appearing to be some type of a ‘natural’ phenomenon, does pose significant theoretical and practical challenges. The potential for miscommunication becomes of increasing import in realms such as scientific, technical, and legal discourse where accuracy can be pivotal. Often, even expert elicitations in such areas may be misunderstood or misrepresented, due to the inadequacy of the spoken word [4,5]. In practical realms such as ground-to-air traffic control in both commercial and military aviation, interpretational ambiguities can and, across the years, have caused disaster [6,7]. Even the use of putatively ‘standard’ operational lexicographies does not eliminate all variation and associated confusion. Our own particular concern for the present issue of communication ambiguity arises from the necessity for precision in language associated with effective interaction between humans and machines, in our laboratory this has most often been expressed as instructions to robots. Such concerns are on the rise, especially with the growth of language-mediated automated and autonomous systems [8]. Many current commercial voice-controlled forms of automation (e.g. ‘Alexa’) can produce evidently adverse outcomes when language terms become confused. While such misunderstandings may occasionally produce benign and even comedic results, the need to accurately interpret meaning is imperative across widespread domains, even extending to academic discourse itself. Often, even terms of common usage are interpreted differently in varying domains of research or disparate operational theaters of practice [9]. As a result of its precision and utility mathematics is often conceived of as being the foundation for terminological accuracy. However, human discourse, and even the common explications of science itself is promulgated, most often, via language. Sharpening the tool of language should therefore be a major aim for all of science, as well as for the improvement of general public discourse in the wider world beyond. Psychological investigative techniques permit us to address this problem, and such an investigation is reported here.

Concerns with the precision of language have been expressed since some of the earliest work on language scale development [10]. Such studies are difficult to conduct and interpret because they involve a complex interplay between various situationally-based responses. The latter are themselves embedded within contemporary and vestigial forms of human response capacities that have accrued along the line of our evolution (see e.g., [11]). Within the basic premises of psycholinguistics, but also expressed in debates upon foundational epistemology, early deliberations sought to address intrinsic philosophical concerns for the communication of meaning. More recently, empirical and qualitative investigations into this specific issue have been reported. This is most notably so in work done to address uncertainty, risk, and error associated in judgement and decision-making [12,13].

**Fig 1:**
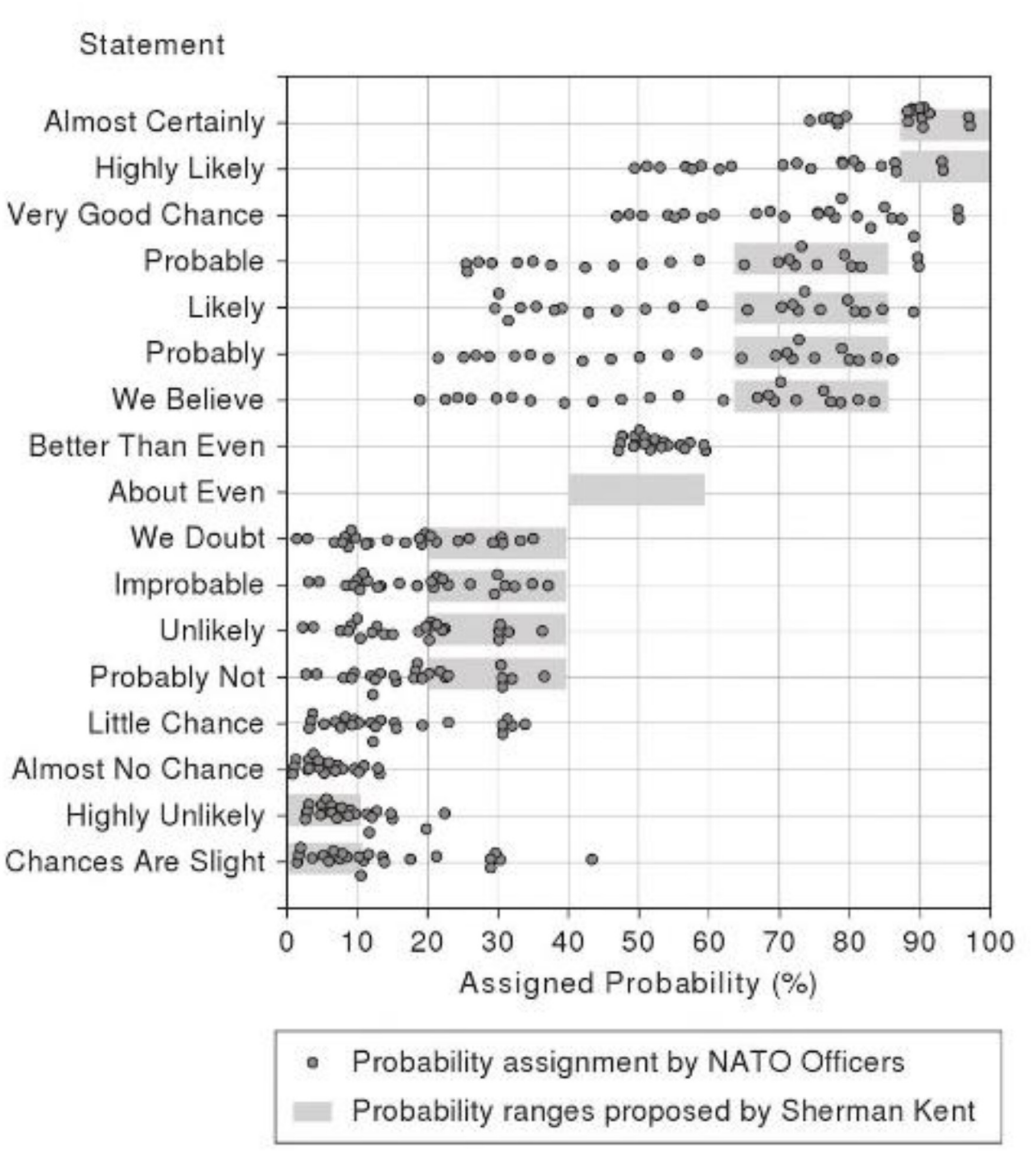
Measuring perceptions of uncertainties. Source: Kent, S. (1994). Sherman Kent and the Board of National Estimates: Collected Essays. History Staff, Center for the Study of Intelligence, Central Intelligence Agency.

**Fig 2:**
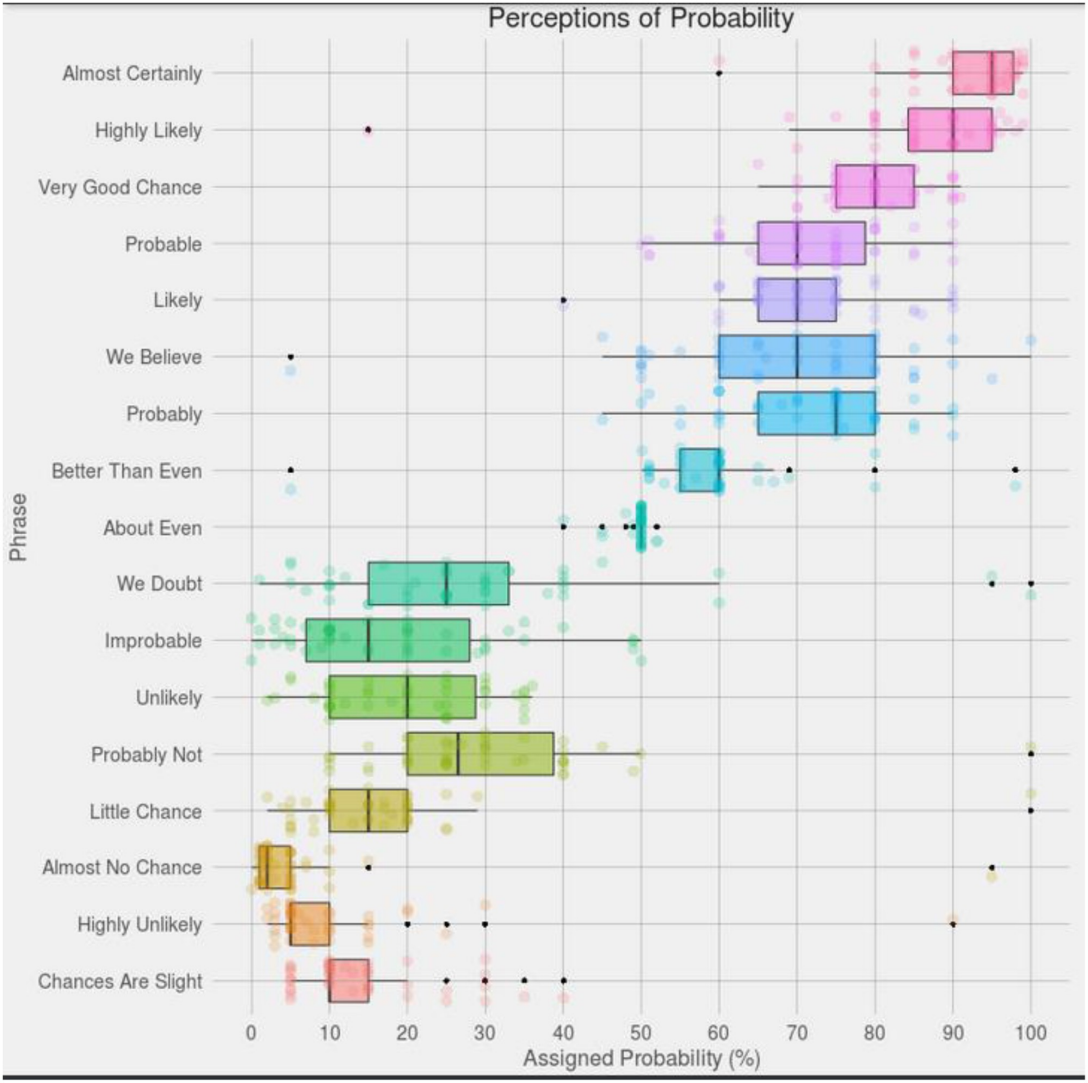
Probability term estimations. Source: Posted to the media website ‘reddit’ by author ‘zonination’ 2015 https://www.reddit.com/r/dataisbeautiful/comments/3hi7ul/.

The history of specific quantitative investigation in this overall domain began, arguably, in the mid 1960’s in an organization that we might not readily connect with the precision of language, i.e., the Central Intelligence Agency (CIA). The author of these CIA reports, and a pioneer in this field, was Sherman Kent. He had originally been a history professor but left Yale to work in Intelligence during WWII. His employment continued for more than a decade and a half, extending into the Cold War era. During this latter epoch Kent helped found the new CIA, Office of National Estimates (ONE). During its active years, this Office prepared more than 1,500 National Intelligence Estimates, aimed at creating a “*literature of intelligence*.” The aspiration was to provide a formal mechanism for the ever-more accurate transfer of knowledge between the analysts and all subsequent consumers of that intelligence. While tranches of these intelligence reports remain classified even today, some works of interest have since been de-classified and have now been released to the public. One of these works, which was classified up until 2007, investigated the variability of aircraft pilots’ estimations of probability terms, [14]. Related to the reliability of intelligence estimates during the Cuban Missile Crisis, this work demonstrated a surprisingly large degree of variability, even within this small and homogeneous sample of professional airmen (Fig 1). As evident in the illustrate data, some terms (e.g., the term ‘probably’) produced estimates that range from a low of 20% to a high of 85% in terms of probability strength, contingent upon the specific individual providing that estimate. Other probability terms showed similar patterns of variation (and see [15–18]). In addition to intelligence-based and research works, other less formal investigations, not published in standard scientific sources and thus not subject to peer review, have nevertheless reached similar conclusions (see Fig 2) [19].

In comparing these respective data sets, which are both derived from the intelligence domain [14], as well as more recent evaluations [19] we witness terms that are rated with almost identical distributions. For example, the term “probable” has a range of 65–85% in Fig 1, and the same term ranges from 65–80% in Fig 2. Similarly, the phrase “probably not” has a range from 20–40% in both data sets. Interestingly however, some terms show evident inconsistencies. The phrase “chances are slight” ranged from 0–10% in Kent’s original work, but was subsequently evaluated from 10–15% in the more recent set of observations. While Kent was able to describe variations among a fairly small and homogeneous sample of U.S. Air Force pilots, in the work we present here we elaborate this effort by examining a far greater number of individuals from a much broader range of backgrounds in order to evaluate whether these reported attributions are constrained or ubiquitous.

**Fig 3:**
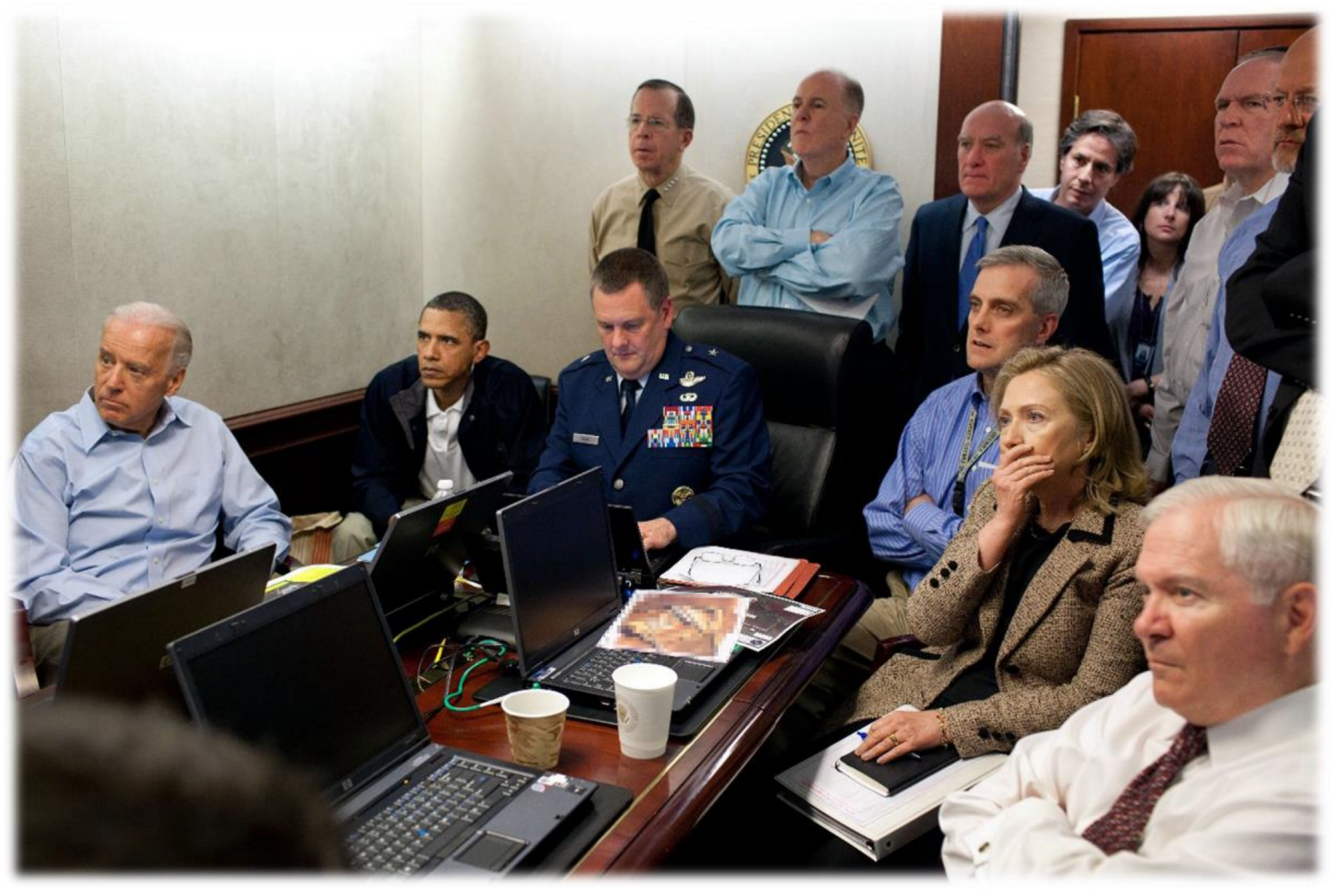
Inside the situation room, May 1, 2011. President Obama, Vice President Biden, Secretary of State Clinton, and others watch in real time the raid on Osama bin Laden.

While the impact of such estimate discrepancies might initially appear somewhat mundane, the reality is that the variability between individuals can easily lead to significant problems. Most especially in situations where accurate understanding proves critical. A well-known example can be extracted from the search for Osama Bin Laden. Throughout the spring of 2011, intelligence estimates on the probability that Bin Laden was living in Abbottabad, Pakistan ranged from 30–40% on the low end, up to 95% on the high end. The National Intelligence Council had spent years producing such intelligence estimates in order to help reduce the ranges of associated uncertainty in communication [20]. Yet these instances of quantitative discrepancy were said to have been “*confusing and even misleading*” by President Obama himself [21]. The final act, which followed this communicated information, proved both distressing and impactful; the effects of which can evidently be seen in the faces of those involved in Washington (Fig 3). This situation was not distressing solely due to ambiguity in communication about risk discrepancies. However, it does highlight the importance of uncertainties in the language used to express the bases for subsequent, critical actions. Thus, for example, the self-same form of doubt and anxiety is evident in photographs of President Kennedy during the Cuban Missile Crisis whose uncertainties triggered Kent’s initial explorations. In these national security briefings, language is overwhelmingly used as the primary conduit of relaying crucial information. Should such information be misrepresented, misunderstood, or mistrusted, dire outcomes threaten to occur. And, of course, inaccuracies in language percolates across all human realms; from marriage to diplomacy and from religion to science.

The foregoing discussion on probability representations clearly describe issues in language communication in general. But similarly, language is also dominant in specific scientific contexts, most especially in the solicitation and quantification of subjective states. The use of assessment tools, such as Likert-type scales, are frequently employed to evaluate these various cognitive affects. Such assessments are employed extensively in contemporary marketing as embedded in modern social media. However, these response scales very often rely on language anchors to gauge relative quantity. A large degree of variability can exist between individuals when interpreting such language terms. This leads to direct questions as to how this variability affects such cognitive assessment tools and the results they produce.

Along with the individual language terms themselves, the layout and spatial distribution of words on scales, such as those that occur on Liker-based constructs, can induce systematic biases [22]. Despite these potential confounds, the Likert Scale is a highly popular survey instrument used in many applications, and has long represented the modal method of attitude specification. Although this investigational approach has been around since the early 1930’s [23], we look here to provide a quantitative evaluation of the numerical accuracy of such scale terms. Our aim in this component of our investigation, being to provide a more accurate degree of numerical accuracy for such scales.

Previous commentators have provided some critiques of Likert-type methods, and how researchers may be able to refine them [24,25]. Such areas of interest being the evaluation of response scale length [26,27], verbal anchoring [28], as well as test design [29]. The typical terms that are used often contain combinations of words expressed as end-points. Anchor dyads such as “strongly disagree,” through “disagree,” to neutral, and then to “agree,” and finally “strongly agree,” are typical designations. These terms are almost always presented using an even physical spacing across a series of selectable choices. Such scale configurations are also most frequently delineated by visual check marks. This way of eliciting response, we argue, does not provide an accurate representation of the true psychological meaning of these terms. These issues, and other potential ambiguities in all linguistic scales, call for a critical re-evaluation of the presentation of such investigative questionnaires. This is important as the results elicited from traditional scale presentations may differ entirely from that of a more veridical representation of expressed choice [8]. More generally, our procedure calls into question the ways we seek to render private psychological experience public in order to engage in subsequent open, quantifiable, inspection.

In the present work then, we evaluate such differing perceptions in these two key areas of language. First, we examine an extensively expanded list of probability language terms that include all those that Kent originally evaluated. However, here we have added further, and additional terms well beyond Kent’s original, restricted number of probability labels. Our goal here being to create a comprehensive spectrum of identified terms, which range from probabilities of 0% to 100%. We then take these elaborated findings to explore linguistic labeling in an applied setting, to reveal disparities in the use of critical language anchors used in survey assessment approaches. This latter evaluation is especially important in affective research as these forms of assessment scales are almost ubiquitously used to render private cognitions open for public inspection.

## Probability descriptors

While probability is a mathematical construct, it is often described via the use of subjective, linguistic labels. Terms such as possible, probable, likely, or a good chance of, are frequently employed in colloquial ways in everyday parlance. In using such terms, there often arise differential percepts as to how likely an event may be when that specific term is employed. This happens in multiple domains and one of the more obvious examples being in risk perception and risk communication [30]. Wallseten and colleagues [31] described two key reasons why we might choose to communicate through such probabilistic expressions. Namely, first that opinions are not precise in and of themselves, and secondly that people feel they are better able to understand words as opposed to numbers. The linkage between language sophistication and numeracy being a well-studied but still cautious issue itself [32]. At first glance, we may suspect that variation in percepts and associated linguistic terms might perhaps make little difference. This might especially be so when qualifying words or phrases or more elaborate discourse can be employed to clarify and mitigate any misunderstanding that might accrue. However, such variations almost necessarily result in problems of ambiguity, partly as a result of the pure frequency rate of their usage. For example, the Intergovernmental Panel on Climate Change (IPCC) have adopted verbal descriptions of probability that are then used to inform policy makers. Yet these may well offer little precision in accuracy and understanding. Misinterpretations are then viewed as leading even to existential threats [33,34]. Additionally, during emergencies in which fast and accurate communication can mean the difference between life and death, miscommunication is often identified as a causal factor in the chain of adverse event etiology, and is a mediator of the damage and destruction that ensues [35].

As a consequence of these potential, and actually realized drawbacks, there have been efforts made to better understand and utilize probability expressions in numerical form. One such example of this effort was the "Good Judgement Project" that most notably studied probability expressions and responses in the context of geopolitical forecasting [36]. Here Moore and colleagues demonstrated the ability of some people to produce useful numerical probabilities, while also reporting high degrees of confidence in their predictions. Probability expressions can also include modifier terms that alter the magnitude of any associated estimate. Work in the field of computer science (CS) for example, has looked to identify the quantification of these modifier terms, based on their association and co-occurrence with other similar terms [37]. These value modifiers are used to create prediction vectors for which terms are likely to be used, given the occurrence of certain key words. Such vectors rely on the associated quantitative value of all utilized terms. Similarly, overall semantic relationships have been studied in CS in an attempt to better understand the linkages words have with one another, both in magnitude as well as meaning [38,39].

**Fig 4:**
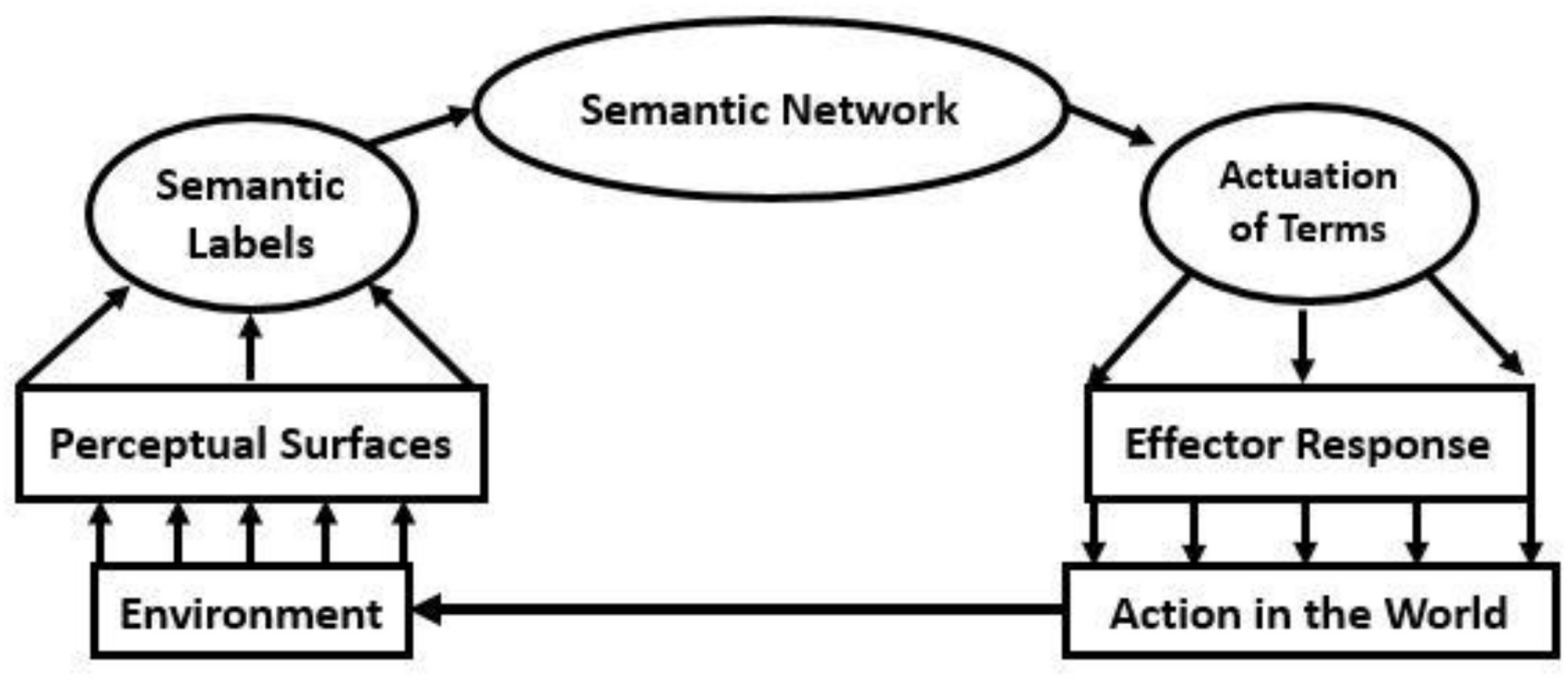
An illustration of the perception-action loop as mediated by sematic referencing systems. Here, the direct perception-action linkage is modified by semantic and qualitative mediations. The imperative of semantic assessment grows as a function of situational complexity and the insufficiently and ineffectively designed technology that mediates across the loop.

Such modeling of the meaning of words and their connections is not solely computational in nature. Theories of perceptual knowledge and understanding have also taken into account the lexical representations we use to describe and interpret the world. For example, Barsalou [40] developed such a model and some his postulations are here incorporated into our own graphical representation of the overarching relationship between perception and action, as mediated by semantic intermediaries (see Fig 4). As these same general forms of assessment also subsume risk analysis, probabilistic terms, and their quantification, are directly pertinent to a wide spectrum of safety operations [3,41,42]. The same issues and concerns also underlie many of the operations in emerging cognitive system engineering, which are largely comprised of complex socio-technical context analysis [2,8,43]. In short, the accuracy of linguistic communication is a vital dimension of many if not almost all forms of human interaction between individual and groups of humans and the technologies they create.

Based upon these various and established premises, our present set of studies into the quantitative spectrum of probability terms is aimed at specifying the degree of variation that such terms elicit and how this affects the way individuals process and communicate using everyday phrases. In particular, the description of rare events often produce seemingly similar average percepts on behalf of a collective group. However, these terms are still often attributed very different quantitative values by each of the individuals within that group who are using them. Our goal is to distinguish quantitative spectra of linguistic terms, initially for “rare” events. Also, we examine the valence, or emotional affect of each term that we also record. This, with the aim of distinguishing quantitative differences due to such positive, neutral, and negative valence attributes.

### Experimental methodology

This study was reviewed and approved by the University of Central Florida’s Institutional Review Board. Approval number: SBE-14-10784. Data was collected online anonymously, thus consent was given by reading an informed consent and choosing to continue participating in the study.

#### Experimental participants

Participants (N = 326, 66% Female, 34% Male, Mean Age = 22.34, SD Age = 5.39) were recruited from the student body of a large southeastern university in the United States. The sample consisted mainly of undergraduates, identified through an online recruitment tool (SONA).

#### Experimental materials

The experiment was deployed via Qualtrics; an online survey tool. Before any data collection participants read and completed an informed consent document that accorded with the guidelines of the American Psychological Association (APA) policy on human participants as well as following the requirements of the current University’s Institutional Review Board (IRB). This process applied to all procedures reported here. Participants then completed a demographic questionnaire, followed by a personality inventory. Demographic information included age, gender, and ethnicity. Personality was assessed via the mini International Personality Item Pool (IPIP) [44]. The “mini IPIP” is a shortened version of the “International Personality Item Pool” and is designed to measure the “Big Five” personality traits of an individual [45]. The Big Five consists of the following dimensions, i) extraversion, ii) agreeableness, iii) conscientiousness, iv) neuroticism, and v) intellect. After completion of these two questionnaires, participants were given blocks of questions in which they were asked to rate the various words that are identified below.

#### Experimental design

**Fig 5:**
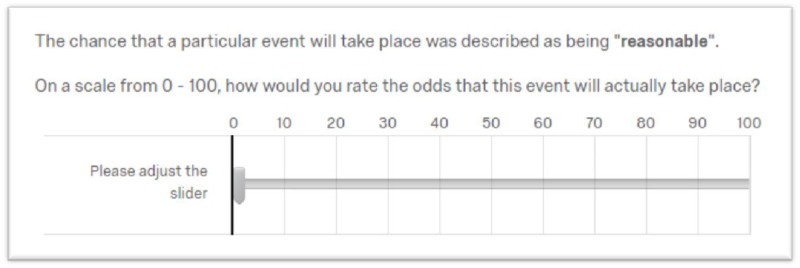
The computer-based sliding scale. Employed in all of the present sequence of experiments to elicit numerical estimates of probabilistic terms.

Participants’ perceptions of identified words and/or phrases were measured via a graphical user interface (GUI) (see Fig 5). Participants were asked to rate the target word or phrase on a sliding scale. For each term, participants were asked to rate the probability of an event occurring from a score of 0 to 100. The sliding scale also contained reference values at every ten point intervals, effectively creating ten deciles in which responses could fall (see Fig 5). This was done to accord with the notion that response reliability has been shown to decrease when greater than 10 response options are used (see [46]). No particular events were specifically stated in association with presented terms. This was done as a deliberate effort to keep the meaning and interpretation of participants’ responses solely on the target word. So, here, any “event” in question was left to the interpretation of the participant.

The precise response procedure consisted of participants first clicking on a numerical location on the sliding scale in the location of their response choice. They were then able to adjust that location, if they felt they needed to do so. They were free to change their selection until they were confident that their precise choice was registered. They were then asked to complete that response via a second confirmatory mouse click (i.e., with the prompt “please confirm your choice”). This process was designed to ensure that any spurious or inadvertent responses were minimized to the greatest possible degree. We endeavored to ensure participants took all the time they needed in order to report the exact response of their choice. This alongside eliminating any error due to inadvertent mouse clicks. Terms were presented in a random order, so as to mitigate any order effects to the degree that this is possible [47]. Additionally, check questions were included throughout the sequence of queries in order to ensure participants were fully engaged in their response. These check questions asked participants to respond with a specific numerical answer (i.e., “please select the value 30 for this question”). Given participants were engaged in the questionnaire, they would answer these specific questions accurately. Participants who incorrectly answered these check questions or failed to complete the survey were excluded from subsequent analysis. A total of 33 participants were so removed due to these violations.

### Experimental results

#### Spectral distribution

We first examined the mean values, representing the average of the reported probabilities from 0 to 100 of an event occurring, when described by each of the candidate words. As illustrated in Table 1, this analysis shows each of the precise terms, its mean value as well as its associated standard deviation (note, Table 2 ranks these terms by those same standard deviation values). Table 2 also reports the number of participants contributing to the estimate and the designated valence of the word itself. Here, we have illustrated these results by ‘banding’ each of the collective terms through progressive decile intervals. This shows that the terms themselves tend to coalesce around their descriptive probabilities in a seemingly face value way (i.e., the term ‘always’ falls within the highest decile on the spectrum) as might be expected a priori. However, it also indicates that terms of lowest probability, e.g., ‘impossible,’ and ‘never’ result in estimates that come closer to their end-point constraint, i.e., zero, than the comparable higher probability terms, e.g., ‘always,’ and ‘certain’ come to their own opposite, maximum anchor of 100 (see Table 1). The identified mean values were subsequently used to develop a spectral model of all probability terms. This sequence ranged from, “always” at the upper bound (M = 85.34, SD = 26.41), being the highest end of the spectrum, to “impossible” at the lower bound (M = 10.60, SD = 20.59). Interestingly, as we have noted, these results show a distinctive narrowing or compression across the original 0–100 range. While *a priori*, intuition would suggest that a spectrum that ranges from “impossible” to “always” should and would translate directly to 0% and 100% anchors respectively, our results demonstrate that the empirical data do not map directly to such pristine expectations. This distortional effect should be a major concern to those who adopt the traditional, assumed mapping when employing these types of data to support broader societal pronouncements [48]. This is an issue that we return to in our subsequent discussion below. The overall outcome for the mean values represents a compressed range from 11% to 85% (cf., Table 1).

**Table 1:** Quantitative distribution of qualitative probability labels. (Ranked by Mean).

	Terms	Mean	SD	N	Valence
1	Always	85.34	26.41	326	Positive
2	Certain	79.82	27.97	325	Positive
3	Almost Always	79.26	24.07	320	Positive
4	Almost Certain	78.17	23.91	324	Positive
5	Very High Probability	77.47	25.06	318	Positive
6	High Probability	76.58	23.52	322	Positive
7	High Chance	75.25	22.33	321	Positive
8	Very Often	72.92	22.84	322	Positive
9	Very Probable	72.41	22.78	317	Positive
10	Very Likely	72.37	22.56	321	Positive
11	Very Frequent	71.45	24.79	324	Positive
12	Often	67.65	19.37	322	Positive
13	Frequent	67.12	20.53	322	Positive
14	Likely	66.71	19.26	325	Positive
15	Usually	65.77	21.56	324	Positive
16	Probable	61.27	21.26	323	Positive
17	Liable To Happen	57.40	23.88	323	Positive
18	More Often Than Not	57.19	20.80	322	Positive
19	Better Than Even Chance	56.05	19.51	323	Positive
20	Possible	53.15	20.48	323	Neutral
21	Moderate Probability	51.23	17.13	324	Neutral
22	Even Chance	48.70	15.38	319	Neutral
23	Not Infrequent	47.89	23.71	318	Neutral
24	Might Happen	46.61	18.81	321	Negative
25	Not Unreasonable	44.61	20.59	317	Neutral
26	Sometimes	44.29	16.16	324	Neutral
27	Occasionally	43.03	17.69	324	Neutral
28	Now And Then	41.03	16.86	324	Negative
29	Every So Often	40.66	18.50	322	Negative
30	As Often As Not	40.52	20.70	315	Neutral
31	Once in a While	33.79	18.63	324	Neutral
32	Less Often Than Not	31.43	17.53	321	Negative
33	Less Than Even Chance	31.42	17.41	320	Negative
34	Unusually	28.44	21.88	321	Negative
35	Not Often	24.88	18.70	325	Negative
36	Not Very Often	24.32	16.54	319	Negative
37	Seldom	23.78	18.88	320	Negative
38	Infrequent	23.43	16.07	322	Negative
39	Unlikely	22.34	18.12	317	Negative
40	Low Chance	22.21	16.91	322	Negative
41	Very Infrequent	21.96	20.54	320	Negative
42	Low Probability	21.56	16.78	321	Negative
43	Very Seldom	21.56	19.73	324	Negative
44	Very Improbable	21.23	23.00	315	Negative
45	Remote	20.65	18.72	320	Negative
46	Poor Chance	19.53	16.44	322	Negative
47	Improbable	19.51	19.28	317	Negative
48	Very Unlikely	19.38	19.04	320	Negative
49	Once in a Blue Moon	19.17	18.37	321	Negative
50	Rarely	19.07	17.98	320	Negative
51	Very Low Probability	18.31	17.89	320	Negative
52	Very Rarely	17.36	19.63	321	Negative
53	Almost Never	14.14	18.99	323	Negative
54	Never	11.30	22.22	286	Negative
55	When Hell Freezes Over	10.91	20.15	292	Negative
56	When Pigs Fly	10.60	20.35	297	Negative
57	Impossible	10.60	20.59	294	Negative

**Table 2:** Quantitative distribution of qualitative probability labels. (Ranked by Standard Deviation).

	Terms	Mean	SD	N	Valence
1	Certain	79.82	27.97	325	Positive
2	Always	85.34	26.41	326	Positive
3	Very High Probability	77.47	25.06	318	Positive
4	Very Frequent	71.45	24.79	324	Positive
5	Almost Always	79.26	24.07	320	Positive
6	Almost Certain	78.17	23.91	324	Positive
7	Liable To Happen	57.40	23.88	323	Positive
8	Not Infrequent	47.89	23.71	318	Neutral
9	High Probability	76.58	23.52	322	Positive
10	Very Improbable	21.23	23.00	315	Negative
11	Very Often	72.92	22.84	322	Positive
12	Very Probable	72.41	22.78	317	Positive
13	Very Likely	72.37	22.56	321	Positive
14	High Chance	75.25	22.33	321	Positive
15	Never	11.30	22.22	286	Negative
16	Unusually	28.44	21.88	321	Negative
17	Usually	65.77	21.56	324	Positive
18	Probable	61.27	21.26	323	Positive
19	More Often Than Not	57.19	20.80	322	Positive
20	As Often As Not	40.52	20.70	315	Neutral
21	Not Unreasonable	44.61	20.59	317	Neutral
22	Impossible	10.60	20.59	294	Negative
23	Very Infrequent	21.96	20.54	320	Negative
24	Frequent	67.12	20.53	322	Positive
25	Possible	53.15	20.48	323	Neutral
26	When Pigs Fly	10.60	20.35	297	Negative
27	When Hell Freezes Over	10.91	20.15	292	Negative
28	Very Seldom	21.56	19.73	324	Negative
29	Very Rarely	17.36	19.63	321	Negative
30	Better Than Even Chance	56.05	19.51	323	Positive
31	Often	67.65	19.37	322	Positive
32	Improbable	19.51	19.28	317	Negative
33	Likely	66.71	19.26	325	Positive
34	Very Unlikely	19.38	19.04	320	Negative
35	Almost Never	14.14	18.99	323	Negative
36	Seldom	23.78	18.88	320	Negative
37	Might Happen	46.61	18.81	321	Negative
38	Remote	20.65	18.72	320	Negative
39	Not Often	24.88	18.70	325	Negative
40	Once in a While	33.79	18.63	324	Neutral
41	Every So Often	40.66	18.50	322	Negative
42	Once in a Blue Moon	19.17	18.37	321	Negative
43	Unlikely	22.34	18.12	317	Negative
44	Rarely	19.07	17.98	320	Negative
45	Very Low Probability	18.31	17.89	320	Negative
46	Occasionally	43.03	17.69	324	Neutral
47	Less Often Than Not	31.43	17.53	321	Negative
48	Less Than Even Chance	31.42	17.41	320	Negative
49	Moderate Probability	51.23	17.13	324	Neutral
50	Low Chance	22.21	16.91	322	Negative
51	Now And Then	41.03	16.86	324	Negative
52	Low Probability	21.56	16.78	321	Negative
53	Not Very Often	24.32	16.54	319	Negative
54	Poor Chance	19.53	16.44	322	Negative
55	Sometimes	44.29	16.16	324	Neutral
56	Infrequent	23.43	16.07	322	Negative
57	Even Chance	48.70	15.38	319	Neutral

#### Valence assessment

**Fig 6:**
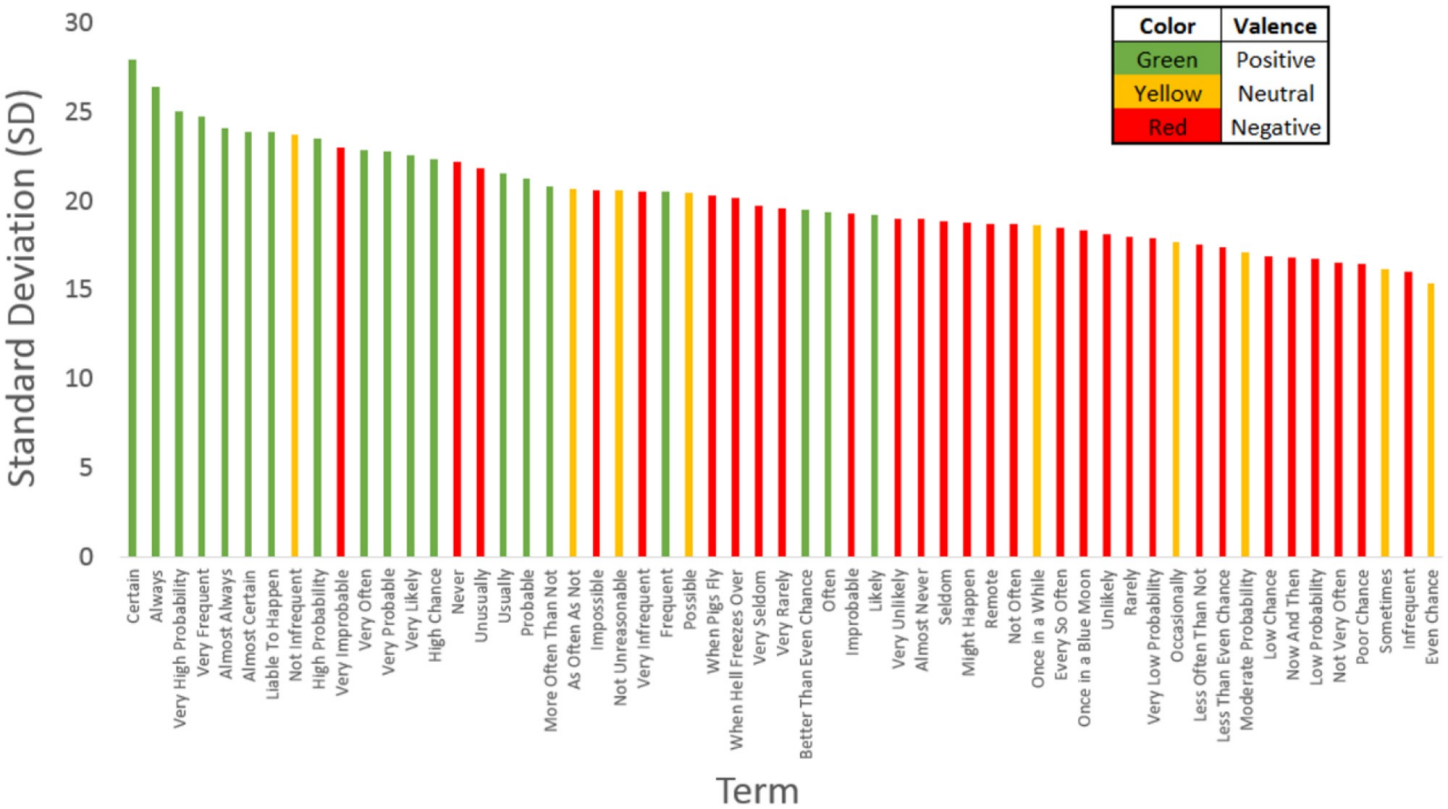
Distribution of standard deviation values against the valence of the expressed word. From this representation, it is clear that words that carry more positive degrees of valence are subject to the larger degree of variability and vice-versa. The interspersed pattern (e.g., ‘very improbable’) however, indicates that this is not simply a function of ordered coefficient of variation as a property of increasing variability with increasing magnitude, but represents a more nuanced differentiation that people’s understanding of number alone.

**Fig 7:**
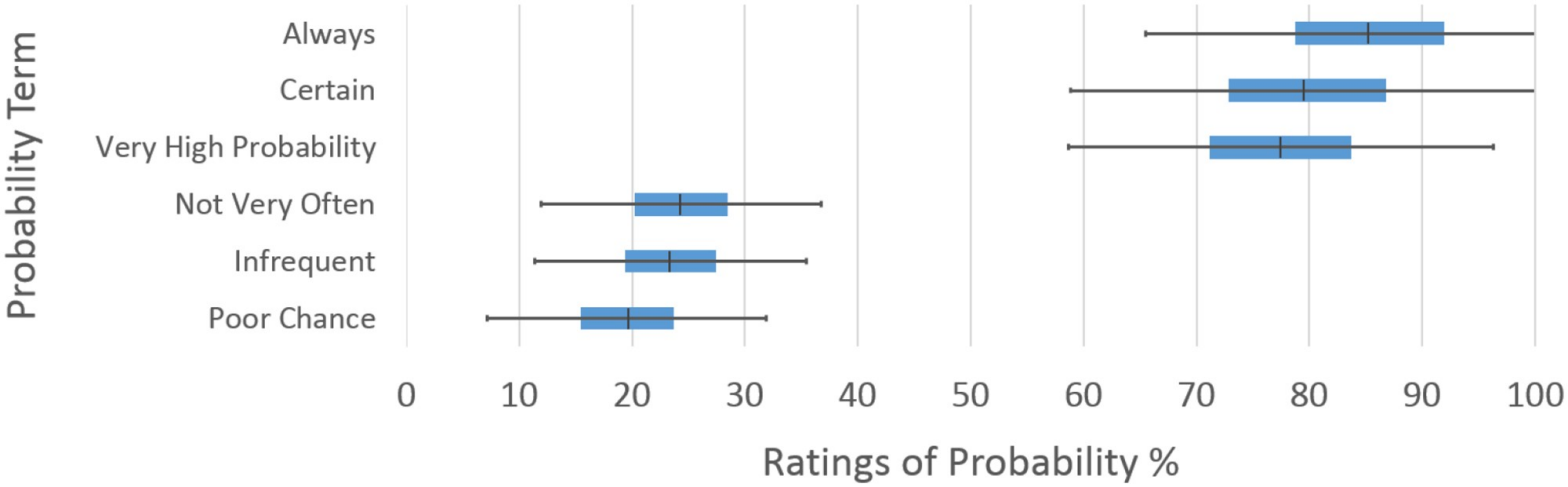
Difference in variance between selected positive and negative valence terms. Larger variation is seen in terms with high ratings of probability, compared to those with lower ratings.

The results in Table 1 also indicate a pattern that is founded on the valence of each specified term. Thus, terms with positive valence, i.e., ones which fell in the upper ends of the valence spectrum, produce greater overall variability across individuals, as compared to the negative valence terms. This effect is illustrated in Figs 6 and 7. Thus, participants provided ratings that were more similar to one another for terms that denoted greater negative affect. These results were also somewhat expected on the basis of foundational numerical understanding. This intuitive understanding of numbers itself does not follow a simple linear relationship with respect to their magnitude (i.e., the understood difference between 1 and 2 is much larger than the difference between 91 and 92) (see [49–51]. Overall however, this pattern of results can still be interpreted to indicate the following. Participants *disagree more on terms that denote events that are likely to happen*, but *agreed more on terms indicating events that are unlikely to happen* (cf., Figs 6 and 7 for illustrations of these terms). Thus, our results demonstrate systematic trends for both valence effects and for the association between magnitude of certainty and associated variability. Given this, we tested the overall correlation between the means and standard deviations of each term. This relationship was found to be significant, *r*(56) = .614, *p* < .001. Such an association indicates that probability terms that were rated with higher mean values also contained greater variance (e.g., “always,” “certain,” etc.), while terms that were rated with low mean values had far less variance among the responses (e.g., “rarely,” “poor chance,” etc.). This result can serve to indicate that the pattern of variability we observed was due to higher means being accompanied by higher variance. Although such data relations have, in the past, been interpreted in theoretical ways; this may also be a statistical property of the process involved in the assessment procedure itself (cf., [32]). However, this pattern has, for example, been found for measures of the summed accuracy of multiple movement trials (see e.g., [52]), as well as for the assessment of sematic variables in general (and see [53]). The same general constraints also apply to reflections of the higher distributional moments beyond mean and standard deviation, i.e., skewness and, to a lesser extent, kurtosis also [54].

#### Individual differences

Additionally, we examined personality factors to assess their influence on individuals’ perception and rating of terms. Overall, positive-valence items were positively correlated with agreeableness (*r* = .168, *p* = .005) and intellect (*r* = .196, *p* < .001). Negative-valence items were, unsurprisingly, negatively correlated with these same personality factors (agreeableness: *r* = -.267, *p* < .001; intellect: *r* = -.216, *p* < .001). These results indicate that people who score higher in agreeableness and intellect are more likely to respond towards the ends of scales. These are general tendencies and the nuances of each individual’s own bespoken choice of numerical rating have yet to be exhaustively identified.

While the uncertainty relative to probability terms and expressions, described above, relate to a general problem in communication, a much more applied question in the domain of lexical ambiguity comes in the form of language anchors for rating scales. Examples such as Likert scales rely on language ratings and are used to compare individual responses, one to another. While there exist many factors such as personality, socio-cultural influence, and applicational domain that influence these mappings to numbers, we have looked here to investigate the specific example of questionnaire response using linguistic labelling. As in probability term ratings, the numerical value represented by each anchor can vary greatly from person to person. This applied context of communication uncertainty is examined in detail in the section that follows.

## Language anchors

Language has sometimes been conceived as being largely subsumed by automated cognitive processes, whereby individuals subconsciously develop their own conceptions of words and phrases. Such individually-mediated and learned calibrations may then lead to idiographic variations in the significance of crucial anchor terms. For example, research has shown that the interpretation of probability expressions are often highly ambiguous [15]. Similar to Kent’s work, these latter authors evaluated a highly homogeneous sample; on this occasion these were male System Development Corporation employees. This group was asked to rate probability on a scale of .01 to .99 of words such as “possible,” “rare,” or “seldom,” etc. While the many of the term ratings were reasonably consistent, asymmetry was found between “mirror-image” phrases (e.g., ‘quite likely’ and ‘quite unlikely’) such that ‘quite likely’ was rated with a mean of .79, whereas ‘quite unlikely’ was rated with a mean of .11, on a scale of .01 to .99 implied probability. In addition, many in the group simply chose not to respond to certain words, and instead reported that they found them too ambiguous to attribute a quantitative probability to. In the field of Human Factors and Ergonomics (HF/E), psychology, and in population sampling in general, behavioral anchors are most often employed in statements of frequency or statements of amount [16]. In the latter work, the authors recorded ratings of thirty-nine expressions of frequency, ranging from “never” to “always”, as well as forty-four expressions of amount; ranging from “none” to “all.” Once again, a high degree of variation was observed between responding individuals. The latter work surveyed a much wider sample, including master’s degree students, adult undergraduates, and high school juniors. Such examples of large degrees of variability in lexical interpretation lead us to question, in the present work, the validity and methodological approach used in rating scales, specifically when used as behavioral anchors.

When such language anchors are employed, descriptions of very rare events produced the most highly disparate probability estimates. Numerical anchors correspond to the ends of any given spectrum of quantitative choices. For example, frequently deployed Likert scales use numerical anchors often ranging between 1–7 or 1–5 to denote the values within the spectral range. Intermediate anchor intervals occur here every one place in these Likert scales (i.e., marks at point 1, 2, 3 etc.). However, linguistic anchoring terms are often (intrinsically) attributed different values by the individuals who use them. As noted, such variations in assumptions as to what is meant by common terms persistently undermine clear and effective communication; and/or on noted occasions, the recorded underlying affective state.

Our specific goal in our second experiment was to investigate the effect related to uncertain terms regarding a variety of different words, and the accuracy of the Likert Scale structure used to present those terms. Relating such terms more accurately to numerical values can provide us with a better understanding of how these words are interpreted across individuals and, of course, by the same individual at different times. Such enumeration can fundamentally change the way surveys and other methods are created, displayed, read, understood, administered and interpreted. These representations would present an opportunity for more accurate identification of meaning and a more efficient manner of gathering data. We further sought to understand whether the propensity to agree with terms of general approbation, and conversely to disagree more with terms of disapprobation, persisted with these specific anchor evaluations. The eventual goal of this present inquiry was to provide a more precise use of qualitative assessment via quantitative values, and thereby further sharpen the tool of language.

### Experimental methodology

#### Experimental participants

Participants (N = 102) were recruited from the student body of a large southeastern university in the United States. They consisted mainly of undergraduates, identified through an online recruitment tool (SONA). Once again, participants were excluded if they failed check questions or if they did not complete the survey. A total of eight (N = 8) participants were so removed from the analysis.

#### Experimental materials

The experiment was deployed via Qualtrics, an online survey tool. Before all collections, participants completed a demographic questionnaire that included age, gender, and ethnicity, followed by the “mini IPIP” personality inventory. Following the completion of these two introductory questionnaires, participants were given blocks of questions in which they were asked to rate the various words that we identify below.

#### Experimental design

Participants’ perceptions of identified words and/or phrases were measured through the use of a graphical user interface (GUI) as previously employed, with one exception noted below (Fig 5). Participants were asked to rate the target word or phrase on a sliding scale relative to the identified term. The exception here was that language anchors were presented on a scale from -100 to + 100, and participants were asked to place the term at the appropriate location on that scale, which contained a zero center point. Once again, after selecting the location of their choosing on the sliding scale, they confirmed their choice through an additional mouse click. Here, our choice was to use a scale ranging from -100 to +100 in order to be coherent with the nature of the stimuli. This also served to alleviate the previously reported correlational effect between mean magnitude and variance in response. As previously stated, the higher the magnitude the more the variance associated. This decision brings with it inherent consequences in comparing negative number assessments with positive number assessments. This is because people often have greater difficulty understanding and rating negative numbers (see [55]). This effect is discussed in relation to the present results below.

### Experimental results

#### Term assessment

We explored the numerical rating differences between commonly used Likert-type scale terms. Table 3 provides a list of these terms and their associated ratings. Here, *as expected*, the valence of the term was directly associated with its rating. All positive valence terms fell above, and all negative valence terms fell below the neutral, (0) value. This pattern is evidently anticipatable from the original construction of such qualitative Likert scales.

**Table 3:** Quantitative representation of Likert scale terms. (Ranked by Mean).

Terms	Valence	Mean	SD
Strongly Agree	Positive	90.0	19.6
Agree	Positive	64.4	35.3
Moderately Agree	Positive	47.0	30.9
Somewhat Agree	Positive	38.5	21.4
Mildly Agree	Positive	32.0	27.3
Slightly Agree	Positive	26.4	26.5
Slightly Disagree	Negative	-14.0	33.1
Somewhat Disagree	Negative	-18.5	36.4
Mildly Disagree	Negative	-22.9	32.7
Moderately Disagree	Negative	-36.9	38.2
Disagree	Negative	-50.6	49.2
Strongly Disagree	Negative	-71.3	56.8

#### Valence distribution

**Fig 8:**
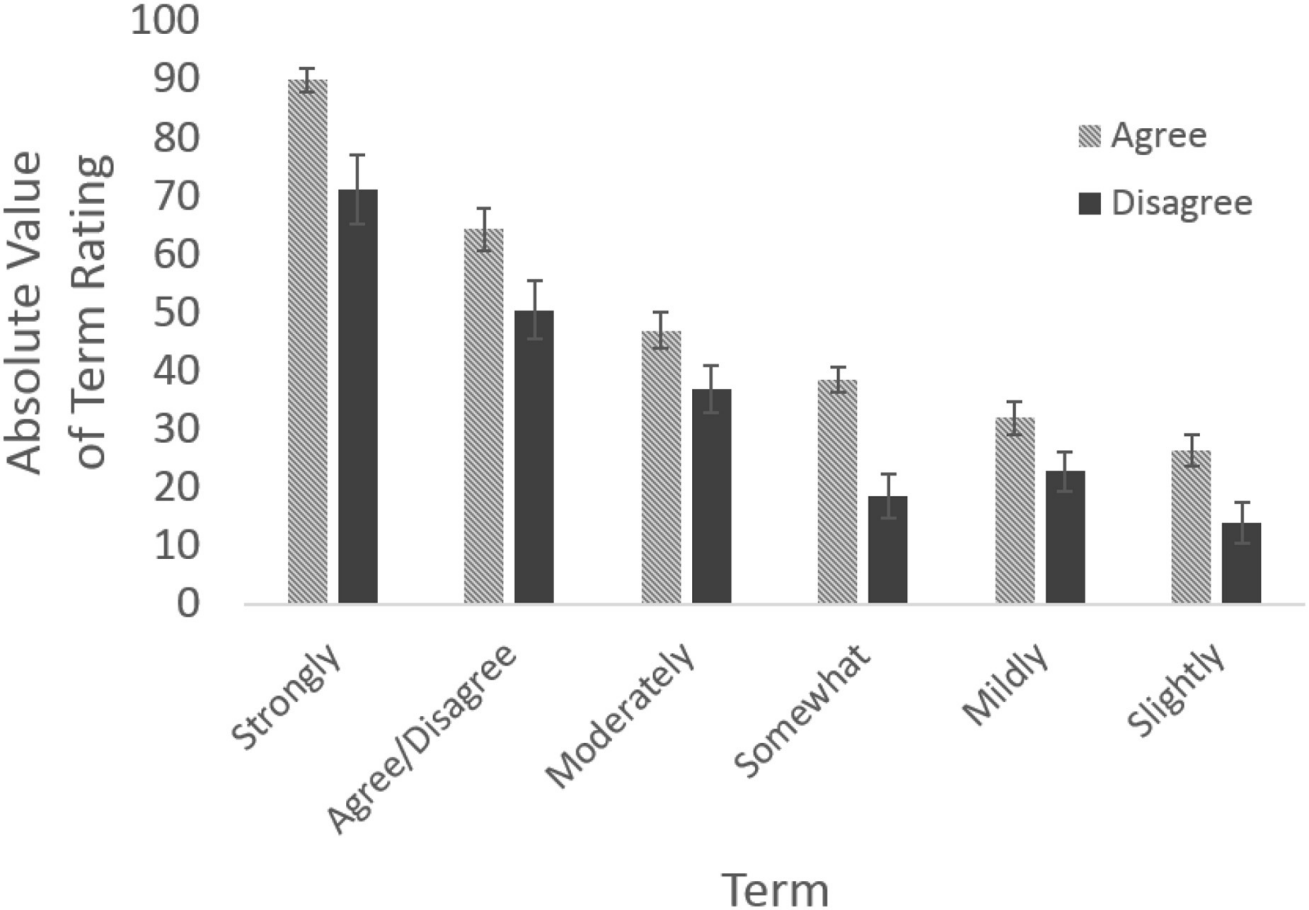
Likert term ratings (depicted here as the absolute distance from 0). Standard Error bars are illustrated.

An important finding here was evident in the overall distribution of means of the ratings. Specifically, all terms exhibited an absolute shift toward the positive. This can be seen for example, in the highest positive term ‘strongly agree’, which was rated at 90.02. In contrast, the lowest negative term ‘strongly disagree’ was only rated at -71.25. This self-same trend can be seen in the pattern of all of the paired terms (i.e., agree-disagree, strongly agree-strongly disagree, etc.). To illustrate this trend more graphically, the negative valence terms have been rectified and are therefore shown as positive values in Fig 8. Here, the shift toward the positive end of the overall possible distribution becomes visibly evident.

Paired-sample *t*-tests were computed in order to provide statistical comparison for each term illustrated in Fig 8. In each case the positive valence term exhibited a significantly higher deviation from the (now) neutral zero point, as compared to their negative valence counterparts (and see also Table 4). Additionally, an overall paired-sample *t*-test was computed that examined the difference between all terms indicating agreement and all those indicating disagreement (transformed into their absolute values). This analysis revealed that overall, terms indicating agreement were further from the neutral 0 point, than terms indicating disagreement, *t*(93) = 3.48, *p* < .001, a finding that is also seen in each individual comparison.

**Table 4:** Significance-values for the paired-samples *t*-tests. Significant differences between positive and negative terms counterparts were found for all term parings.

Term Comparison	*t*(df)	*p*	Cohen’s d
Strongly agree/disagree	3.50 (93)	<.001**	0.44
Agree/disagree	2.23 (93)	<.05*	0.32
Moderately agree/disagree	1.99 (93)	<.05*	0.29
Somewhat agree/disagree	4.32 (93)	<.001**	0.67
Mildly agree/disagree	2.05 (93)	<.05*	0.30
Slightly agree/disagree	2.77 (93)	<.05*	0.41

**Fig 9:**
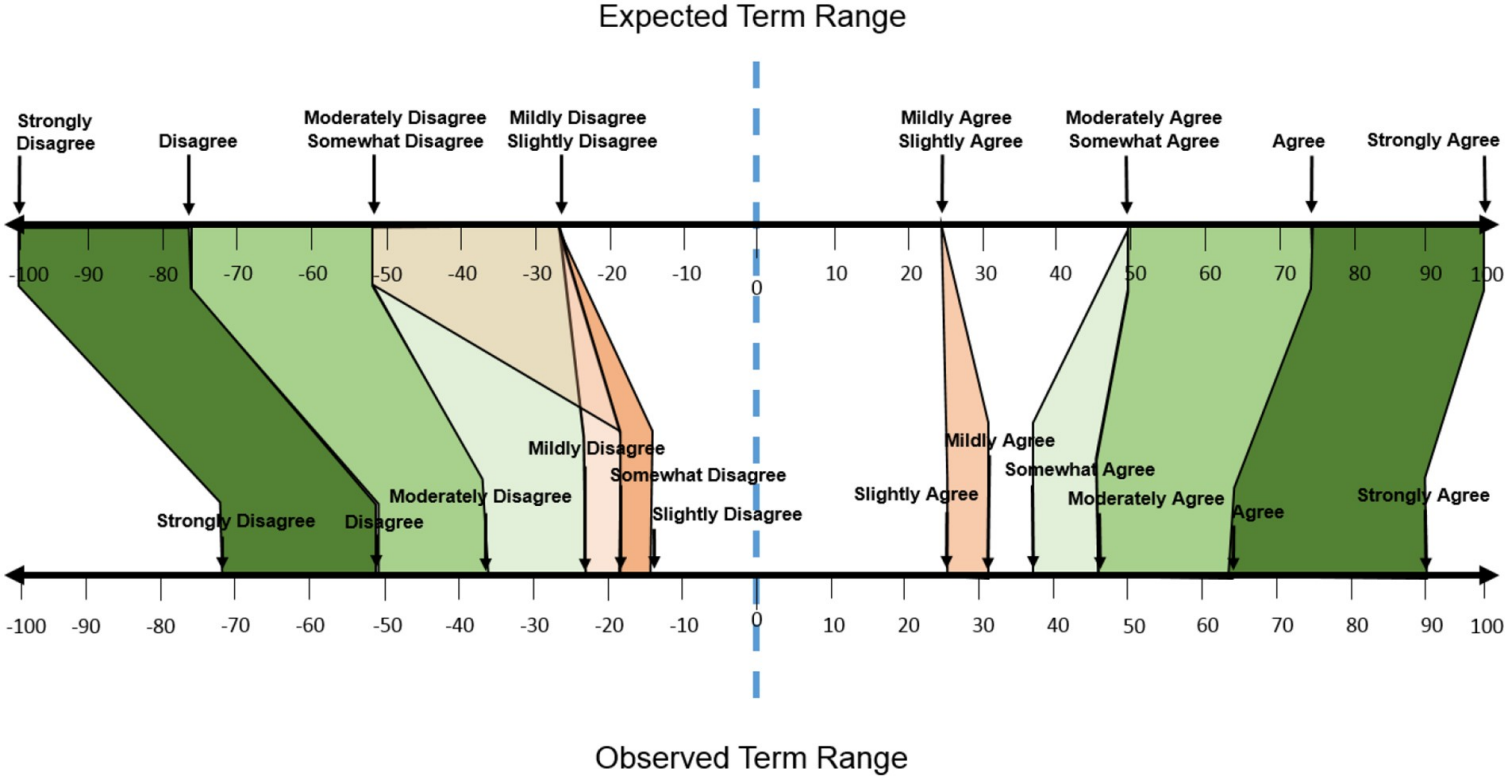
Comparison of the assumed quantitative locations of the theoretical anchor term (top scale) versus the experimentally determined anchor term positions derived from empirical observation (bottom scale). Negative valence terms showed a larger shift towards the center point, as compared with their matched positive valence terms.

We examined the intervals between the Likert-like terms in respect of these results. A priori, it would be anticipated that each of the terms would exhibit a standard interval between each other. However this did not prove to be the case. On the top scale, of Fig 9 is depicted these theoretical locations of the discrete terms along a -100 to +100 scale. Here, each term is assumed to represent an even addition of strength from each of its predecessors, while the collective terms are distributed across the whole of the possible range. This is the assumptive interpretation that is given by psychological subjective measurement indices, including essentially all Likert scales themselves. Despite this widely accepted interpretation, we did not find this assumptive distributional pattern to be the case. Instead we found that the inclusion of terms such as “moderately”, “somewhat”, “mildly”, and “slightly” caused greater compression of the representation than the inclusion of the term “strongly.” Additionally, the main terms themselves (i.e., agree and disagree) did not present even intervals along the interpretational spectrum (see Fig 9).

To address this directly, we performed an analysis on the positive and negative anchor terms respectively. This chi-squared analysis of the positive terms, X^2^(5) = 7.35, *p* > .05, showed that observed values did *not* significantly differ from the expected values. However, the comparable chi-squared analysis on the negative anchor terms, X^2^(5) = 40.92, *p* < .001, did indicate that observed values were significantly different from expected values. These results demonstrate that negative anchor terms show a significant compressive trend towards the center of the scale. Thus, negative expressions here do not represent the expected theoretical values. This finding that positive and negative anchors did not result in similar patterns indicates a clear disparity in the scale, such that overall it significantly skews towards the positive (Fig 9).

Variability values in the present results were once again dependent on the valence of the identified term. Positive valence terms exhibited lower degrees of variability, compared to their negative valence peers. This decreased level of variability may itself have contributed to the overall trend toward the positive. As participants’ ratings aligned more closely on positive valence terms, those terms fell more accurately with respect to the anticipated distribution (i.e., closer toward the end of the spectrum; and see Fig 9). This finding is an interesting contrast to the results of our first experiment. When rating terms on a 0 to 100 scale, we see the largest variation at the top of the scale, however when using a -100 to 100 scale the largest variability is instead seen in the negative range. Once again, this echoes the finding that people often struggle to understand and use negative numbers (see [55]).

#### Individual differences

We again examined certain individual difference factors in order to seek to account for these outcome patterns. Using values obtained from the mini-IPIP for each of the Big Five personality traits, there proved to be no specific traits that mapped directly onto these results. However, we found that participants could be classified along a spectrum of ’strength of response’. Average means for both positive and negative valence term groups were found to significantly and negatively correlate with one another *r*(82) = -.590, *p* < .001. This indicates that individuals tended to respond with similar strengths of response overall. This means that some people respond closer to the neutral (0) point for all responses, while others tended to respond closer to the ends of the spectrum. Unfortunately, as none of the individual metrics assessed here (i.e., personality, age, and gender) mapped on to this pattern, the source of this tendency awaits further elucidation.

When we seek to render private apperception of one’s own personal cognitive state into an external and inspectable form, there are certain intrinsic assumptions about the process by which this is done. This is most especially because of the apparent degree of ‘certainty’ with which these quantitative forms of data are then expressed (i.e., strongly agree, agree, etc.) and interpreted. These transformations from affect to metric also tempt us toward subsequent mathematical transformations and computations based upon these now apparently quantified numerical data. This issue is somewhat reflective of Stevens original observations and concerns about the differing orders of measurement that he identified [56]. We have shown here that the underlying assumption of equivalence between putatively ‘equal’ agreement/disagreement terms is suspect at best and simply wrong at worst. Further, what individuals actually mean by each term is subject to important and differing personal interpretations. Thus, while individuals show quite close agreement about the term “strongly agree” they are almost three times more variable about its antithetical counterpart “strongly disagree”. In essence, *people agree with what they agree about but disagree over their disagreements*. This is a finding that mirrors the same important outcome expressed in our prior experiment [and see also 50]. This pattern is also somewhat supported by the link to the personality trait of agreeableness, which here correlated positively with the term "strongly agree" *r*(94) = .216, *p* = .036. Yet, in comparison, did not significantly correlate with the term "strongly disagree" *r*(94) = -.075, *p* = .470.

#### Scale validity

**Fig 10:**
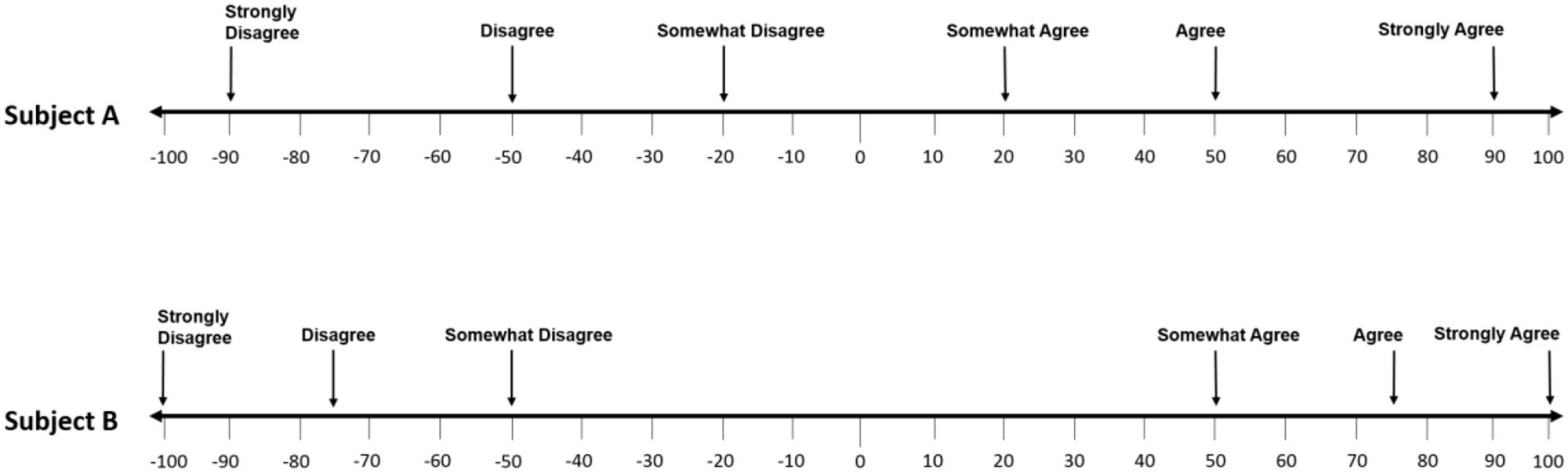
Theoretical comparison of two interpretations of Likert scale anchors. Participant “A” is shown to represent even spacing of term meanings, while participant “B” interprets the terms towards the ends of the spectrum.

While our results clearly demonstrate that numerical rating scales do not necessarily map directly to a pristine interval representation, the high degree of variability between individuals brings into question the validity of using such scales in the first place. Take, for example, two individuals responding on a traditional seven-point Likert scale. To each participant, the value that a given term represents can be unique. So, while each of these people may still see the scale as ordinal, their perception as to its distribution of terms can be radically different. Such a contrasting propensity is shown for illustrative purposes in Fig 10. In the example illustrated, Subject A views the terms as more evenly spaced across the spectrum. Subject B, in contrast, has grouped the relevant terms according to one particular attribute (i.e., the inclusion of the words ‘agree’ and ‘disagree’). This discrepancy results in manifestly inaccurate comparisons between the expressions of thought derived from these two individuals (see for example their respective placement of the term ‘agree’).

These types of assessment instruments are widely administered, both within experimental psychology, but also across much larger swathe of human society [57]. If we cannot be sure that they translate any affective state homeomorphically to its numerical attribute, or even link consistently to the presented linguistic labels, then evidently opinions expressed do not reflect each person’s real subjective apperception. Worse, when such information is then subject to subsequent numerical transformations then it is more than probable that the answers derived will be not just inaccurate but simply wrong. We are already aware of certain procedural shortfalls in public opinion surveys (e.g., non-random sampling, etc.). However, our objection here is a much more fundamental and profound one. Our present data serve to question the epistemological foundations of all such windows on understanding of the expressions of human cognitive states in the first place.

## Overall discussion

Language, at least in rudimentary forms, has existed for perhaps many millions of years. However, the scientific study of such language is much more recent [58]. These modern form of scientific focus has only really flourished in the past two centuries. It has largely been formalized in psycholinguistics and a variety of allied language study related disciplines. Modern exploration of language from a psychological perspective is derived from foundations established by the likes of Chomsky, Lakoff, and others. These individuals each helped to define the field as it exists today. Recent studies of language, through the advancement of computing power, have evolved into complex analyses of word relatedness and word and term co-occurrences. However, our work represented here maintains primarily a psychological focus, by assessing the reaction of human respondents.

Subjective language assessment is used in almost all areas of psychology, ranging from clinical diagnostic practices to applied human factors [2,59]. While both powerful and accessible, subjective assessment, through the use of language, does not come without its methodological drawbacks and inherent limitations. The present work has explored some of the ambiguities, and how they can bring with them miscommunication and incipient misunderstanding. We have documented that even standard linguistic terms often produce large inter-individual variation, even among putatively homogeneous samples. Our results represent evidence of ‘dissociation’ between ‘objective’ probabilities and the then perceived rates of their occurrence [8,60–62]. While we do not believe that individuals in general, walk around conceiving of sematic terms such as ‘likely,’ directly in terms of quantitative values, the experimenter can ask people to express such ideas using numerical external scales. Human beings can and do however, often use numbers in acts of deliberate cognition, and thus is indeed the topic of a whole, spate area of research concerning the nuances of non-verbal, numerical cognition [see e.g., 63, 64]. The confluence of verbal and numerical forms of cognition may even be especially important in relation to activities in science and technology. Thus, the further confluence of technical and colloquial usage of verbal representations permeates modern society. Inaccuracies can flow back and forth and the result of imprecise language is imprecise action. On many occasions such imprecision is mitigated or dispersed by additional dialogue or other forms of information exchange. On other such occasions such imprecision proliferates and can well result in adverse consequences.

Previous work by Hancock and colleagues [22,65,66] has shown the large variance in the perception of words that occur when using diverse categories of expressions such as “underspecified terms” and “phrases of doubt”. The first experiment in this series identified the variability between individuals in their assessment of words representative of probability. Additionally, evidence that personal differences may play a role in the range of meaning attributed to terms was also established. Here we found the individuals who score high on agreeableness and intellect tend to rate probability terms more towards the ends of the available response spectrum (i.e., lower ratings for low probability terms, and higher ratings for high probability terms). They thus seem to use more of the possible response spectrum then those less endowed with these characteristics. This finding could be used to better equate probability interpretations by examining such personality differences, and adjusting numerical representations accordingly. While these results show individual patterns of response, they are by no means a complete representation as to *why* individuals respond in the manner they do. However, we argue that our information here is critical as the foundations of and stimulus for subsequent exploration and understanding of these ‘individuated’ assessments [see 67].

To re-iterate, our goal in the first experiment reported here was to produce a comprehensive ordering of a spectrum of probability terms, which was indeed achieved (Table 1). While we accomplished our overall goal, the results did not show a range from 0% to 100%. Instead we found a narrowing or compression of the actual spectrum from 11% to 85%. These end points were represented by the terms “impossible” and “always” respectively. These mean levels were likely due to the large amounts of variance seen in each rating, as well as the tendency of some individuals to consistently report more centralized ratings (i.e., closer to neutral).

It is commonly assumed that rating scale measures themselves are one source of certainty, although we have established here that this is not the case. As we have shown however, such rating scales can themselves produce an intrinsic variance, resulting in un-accounted for variance in vast swathes of such survey results. This intrinsic variance is once again caused by the interpretations of the words used to describe anchor points. Similar to the probability term interpretations, individuals often associate widely different numerical meaning to language terms. We believe this may well be a propensity that extends well beyond the specific terms we have used in the present experiment and so represents a challenge to all others who employ the tool of language. It may be possible to mitigate these effects to some degree by reducing information to conform to ordinal scales only. However, certain transforms cannot be made on ordinal representations and the absence of an absolute anchor or equivalent intervals between terms seems to be more blunting than sharpening the tool of everyday discourse.

Our present findings also show the unequal distribution of strength across valence. This is most especially evident in the second experiment, where we investigated language anchors. Positive valence terms demonstrated a stronger trend to extend further from the center of the scale; thus, being rated as more extreme, as opposed to the negative valence terms that fall more toward the center of the scale. This discrepancy, expressed as an interaction with valence, can lead to evident inaccuracies in associated psychological evaluations, when such terms are specifically intended to match to their opposite valence counterpart.

The specific distribution of terms here was also not found to represent a series of even increments in value. The typical use of scale anchors assumes each choice represents an even step from each of its immediate neighbors. For example, in a seven-point Likert scale the terms “slightly agree”, “agree”, and “strongly agree” are intended to represent discrete and even step increments in choice options. Underlying this principle is the understanding that such choices are representative of the subject’s own state and intended qualitative representation. Our findings however, indicate that these actual discrete choices are not representative of such equally divided depictions. We argue here that a potential future solution would be to provide standard, quantitative values to each of these modifier terms, in association with known populational variability levels. This would facilitate the production of verified numerical representations of such scale ratings, when used across the many realms of subjective assessment. Additionally, this would provide a method of representing such assessments in an interval, or even ratio basis, as opposed to simply an ordinal sequence as is currently the case. This step would help to reduce, or aspirationally eliminate some of the problems inherent to measurement such as mismatched anchors (as seen in Fig 10).

### Limitations

While this paper has addressed some of these inherent issues in lexical interpretations and ambiguities, our work here is not without its limitations. One potential issue with the findings from the second experiment for example concerns the fact that negative valence terms exhibited a shift towards the positive end of the spectrum. This may result in part from inherent difficulties in interpreting negative numbers [see 47]. Specifically, this issue in rating negative numbers could apply here to the interpretation and rating of negative valence terms that used such negative numbers. If individuals had varying degrees of understanding or representation of negative values in general then higher degrees of variance in the negative terms might well be anticipated.

Whether it is a specific limitation or not, one question that ought to be considered is the occurrence and observed persistence in variability of estimates of terms of probability. We have in our evaluation, tended to treat such variability as a ‘problem’ or ‘issue’, but it may well be that such variability presents an evolutionary advantage. For example, the successful functioning of many forms of cybernetic systems appears to be predicated upon the notion of ‘requisite variability’ [68]. Here, such variation proves useful, and even essential to the tuning and operation of feedback-mediated mechanics. From this perspective variation may serve a strong positive purpose, perhaps in encouraging communication and discourse by the use of extended language interchange. As our experiments focus largely on single terms or brief phrases, such as advantage may not have been manifest in considering only limited terms. If imprecision in language is actually truly adaptive it is important to understand further how such a mechanism may work. Regardless, the antithesis to the ‘unwanted’ perspective on variability is always important to consider and it would be remiss not at least to acknowledge this here.

Another remaining concern is that there is evidence that rating scales decrease in their reliability beyond ten identified response categories [69]. Preston and Colman here argue that scales with 7, 9, or 10 response categories are preferred for accuracy and reliability. An argument can be made that the method of response that we used here (0–100) might thus result in decreased reliability, given the large absolute number of possible response integers. However, we would reply to this objection that the sliding scale that we provided (Fig 5) gives the individual the opportunity to scale their response in respect of each individual decile (i.e., every ten points). Additionally, evidence suggests that humans can only accurately employ up to five numbers consistently [70]. However, here participants were asked to rate many terms and were required to use far more than five numbers during the course of each study. Yet, because these response categories are always present on the screen, and not subject to memory limitations [71], the objection as to numerical responses are here largely obviated.

### Conclusion

Lexical ambiguity is not only an issue in verbal communication. It can also induce problems in psychological interpretation and evaluation. Applied technical domains, such as alerts and warnings, require and mandate accurate affective representations and understandings of communicated information [72]. Real-life safety and risk assessment hinge on such accurate, and often implicit understanding. Communication protocols in operationally-critical domains, such as air-traffic control, are often formulaic in a purpose-directed attempt to excise communication ambiguities. Of course, this strategy does not always work [35]. Language ambiguities are also critical in legal proceedings in which the interpretation of a spoken phrase can mean the difference between life and death [73], as they can also inter-national diplomacy [74]. The importance of the present concern cannot thus be denied.

Such problems continue to persist. For example, for the past seven decades that have followed upon the murder of Police Constable Miles in Croydon, England, no one has been able to agree on the exact meaning of the fatefully uttered words, “*let him have it*, *Chris*.” Did the speaker, one Derek Bentley, mean that his teenage partner Christopher Craig should give up his gun, or did he mean Craig should shoot the police officer? The phrase has become a classic example, frequently quoted, to show the ambiguity and criticality of language. It was, in large part, this intrinsic ambiguity that sent Bentley to his appointment with the hangman. Words matter and on many occasions are fateful [75,76].

Often, ambiguity is caused by both the variance in the utterances themselves and in the interpretation of the words in their turn. Such ambiguity is contingent on the initial choice of word or phrase as well as the class of decision it may evoke. Uncertainty in higher risk domains such as finance, healthcare, or international security, may produce vastly different biases and response criteria as opposed to putatively lower level risk areas, such as daily weather patterns or sporting forecasts [77]. In short, while in some domains the use of “a good-enough representation” is adequate, the suitability of this approach to communication is context-specific. What proves ‘good enough’ or ‘satisficed’ in one context [78] often proves problematic in another.

It is also feasible that ambiguity arises from noise inherent in the communication channel between transmitter and receiver. In human intercourse this being direct speech or technologically-mediated interactions. Through the further investigation of such sources of misunderstandings and the ambiguity associated with each, important advances can be made in methods that promote easy and consistent understanding. To move forward, it is important to specify the ambiguities that we see inhibiting the efficiency of what is perhaps the most ubiquitous tool used by the human species; language itself. The specific procedures discussed herein, such as psychological evaluation surveys, do lend themselves to additional investigation and improvement. We therefore continue the effort to develop and refine these psychological evaluation techniques. Thus, in the future, we look to provide a more accurate externalized representation of the operations of the human mind.

## Supporting information

S1 Raw data (XLSX)
